# Intramedullary Gas Detected on Computed Tomography in Cases of Decompression Sickness: A Case Series

**DOI:** 10.7759/cureus.101936

**Published:** 2026-01-20

**Authors:** Tomoko Sugimura, Nanae Tsuchiya, Takumi Chinen, Akihiro Nishie, Takehiro Umemura

**Affiliations:** 1 Department of Emergency and Critical Care Medicine, Graduate School of Medicine, University of the Ryukyus, Ginowan, JPN; 2 Department of Radiology, Graduate School of Medicine, University of the Ryukyus, Ginowan, JPN

**Keywords:** computed tomography, decompression sickness, intramedullary bubbles, intramedullary gas, marrow bubbles

## Abstract

Decompression sickness (DCS) is caused by nitrogen gas bubbles in the blood that lead to bubble embolization and tissue compression. Intramedullary gas bubbles may also be observed; however, their clinical significance remains unclear. Herein, we describe our experiences with three cases that experienced DCS in which intramedullary gas bubbles were incidentally discovered on computed tomography (CT) performed before treatment.

Case 1 involved a 28-year-old male who had repeatedly dived to 30 m and surfaced. After diving, the patient experienced pain in the left lower extremity. CT revealed bubbles in the right atrium, hip joint, and humeral marrow cavity. Case 2 was a 55-year-old male who had dived for 25 min to a maximum depth of 42 m. Thirty minutes after diving, the patient experienced nausea and thigh pain. CT revealed bubbles in the brachiocephalic vein, pulmonary artery, femoral vein, intrahepatic portal vein, and the femoral bone marrow cavity. Case 3 was a 52-year-old female who dived to 50 m before abruptly surfacing. Subsequently, the patient experienced dizziness and nausea. CT revealed bubbles in the mediastinum, intrahepatic portal vein, femoral vein, and brachial bone marrow cavity.

These findings demonstrate that gas bubbles may be observed within the bone marrow on CT scans of patients with mild cases of DCS.

## Introduction

Decompression sickness (DCS) occurs when nitrogen gas bubbles form in the blood and tissues during ascent from a high-pressure environment such as diving or caisson work, leading to gas embolism and tissue injury [1]. The diagnosis of DCS is primarily clinical, based on a compatible exposure history and characteristic symptoms, and treatment consists of immediate oxygen administration and early hyperbaric oxygen therapy (HBOT). Imaging is not required for diagnosis, but may be performed to evaluate complications and exclude contraindications to HBOT, such as pneumothorax. Nitrogen bubbles commonly accumulate in poorly perfused tissues, particularly adipose tissue, and have been reported in the joints, mediastinum, mesentery, portal vein, spinal canal, and cerebral ventricles [2,3]. Although intramedullary gas detected using computed tomography (CT) has been reported previously [4], it may be under-recognized in clinical practice, particularly in patients with mild DCS who do not routinely undergo CT imaging. This case series describes three patients with acute DCS in whom intramedullary gas was incidentally identified on CT scans before HBOT.

## Case presentation

Case 1

A 28-year-old male professional diver (height: 165 cm; weight: 62.5 kg; body mass index (BMI): 22.8 kg/m^2^) presented to the emergency department.

History of Presenting Illness

The patient had repeatedly dived to a maximum depth of 30 m over 90 minutes. The patient used compressed air as the breathing gas during all dives. Upon surfacing, the patient experienced numbness in the left lower limb, followed by bilateral lower limb pain three hours later. The patient denied alcohol use but had smoked 10 cigarettes per day for 10 years and had no prior history of DCS.

Physical Examination

The patient was alert, with a heart rate of 69 beats/min, blood pressure of 102/70 mmHg, respiratory rate of 20 breaths/min, peripheral oxygen saturation (SpO_2_) of 100% on a reservoir mask at 15 L/min, and temperature of 37.6°C. The patient had tenderness in both thighs but no other circulatory or neurological deficits. Transthoracic echocardiography did not demonstrate a patent foramen ovale.

Imaging Findings

**Figure 1:**
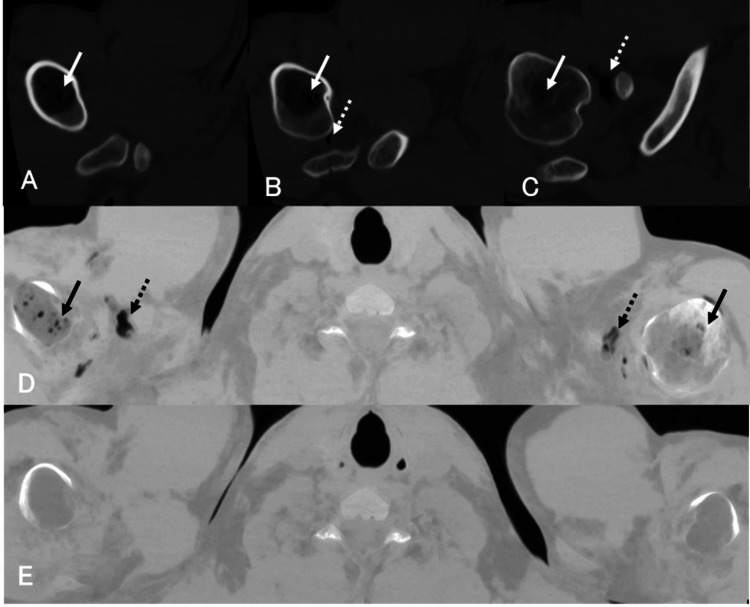
Case 1 A-C: Bone window settings. All computed tomography (CT) images were reviewed using a bone window setting with a window width of 3000 HU and a window level of 400 HU. D-E: Minimum intensity projection (MIP) images. Pre-hyperbaric oxygen therapy (HBOT) plain CT images (A-D) demonstrate multiple gas densities in the proximal humeral bone marrow bilaterally (solid arrows) and the right shoulder joint (dashed arrows). E: Complete resolution of intramedullary gas following HBOT.

CT (Figures 1A-1D) showed gas in the right atrium, right ventricle, bilateral sternoclavicular and shoulder joints, bilateral hip joints, both humeri, and left femoral bone marrow.

Clinical Course

HBOT was administered in accordance with the U.S. Navy Diving Manual Table 5 (a standardized treatment protocol, not a table included in this manuscript). A repeat CT scan on day 4 (Figure 1E) demonstrated complete resolution of the intramedullary gas. HBOT was completed after six sessions.

Case 2

A 55-year-old male recreational diver (height: 174 cm; weight: 92 kg; BMI: 30.4 kg/m^2^) presented to the emergency department.

History of Presenting Illness

The patient had completed a 25-minute dive to a depth of 42 m. Thirty minutes after surfacing, the patient developed nausea, followed by pain in the right shoulder, hip, and thigh. The patient had no history of alcohol consumption or smoking but reported type II DCS with pneumocephalus eight months earlier and had untreated interstitial pneumonia.

Physical Examination

The patient was alert, with a heart rate of 92 beats/min, blood pressure of 131/88 mmHg, respiratory rate of 22 breaths/min, SpO_2_ of 100% on a reservoir mask at 10 L/min, and temperature of 36.7°C. He had pain in the right shoulder, right hip, and left thigh, but no other abnormalities. Transthoracic echocardiography did not demonstrate a patent foramen ovale.

Imaging Findings

**Figure 2:**
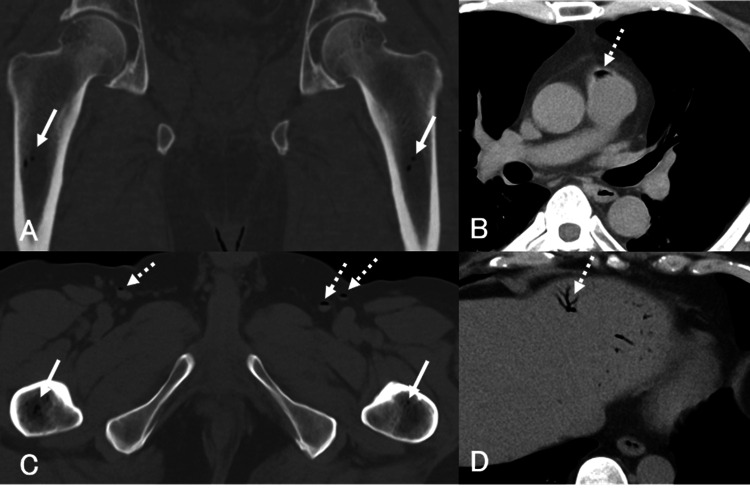
Case 2 Pre-hyperbaric oxygen therapy (HBOT) plain computed tomography (CT). A, C: Bone window settings (A: coronal view; C: axial view). All CT images were reviewed using a bone window setting with a window width of 3000 HU and a window level of 400 HU. B, D: Mediastinal window settings. Intramedullary gas is identified bilaterally in the proximal femoral bone marrow (solid arrows). Multiple gas densities are also observed in the pulmonary artery (B), femoral vein (C), and portal vein (D) (dashed arrows).

CT (Figures 2A-2D) revealed gas in the brachiocephalic vein, pulmonary artery, bilateral femoral veins, intrahepatic portal vein, bilateral sternoclavicular joints, and left hip joint. Intramedullary gas was present in the left clavicle and both femurs. Post-HBOT CT scans were not performed.

Clinical Course

HBOT was administered according to Treatment Table 6 of the U.S. Navy Diving Manual. The thigh pain improved by day 3, and HBOT was completed after eight sessions.

Case 3

A 52-year-old female recreational diver (height: 163 cm; weight: 82 kg; BMI: 30.9 kg/m^2^) presented to the emergency department.

History of Presenting Illness

After a 45-minute dive to 10 m, followed by a one-hour surface interval, the patient resumed diving. The patient panicked at 50 m and developed dizziness and nausea during the ascent. The patient denied alcohol or tobacco use and had no history of DCS.

Physical Examination

The patient’s mean Glasgow Coma Scale score was 15. Vital signs included a heart rate of 80 beats/min, blood pressure of 139/102 mmHg, respiratory rate of 22 breaths/min, SpO_2_ of 100% on a reservoir mask at 15 L/min, and temperature of 36.2°C. The patient reported dizziness, nausea, vomiting, and tinnitus but had no musculoskeletal pain or neurological deficits. Transthoracic echocardiography did not demonstrate a patent foramen ovale.

Imaging Findings

**Figure 3:**
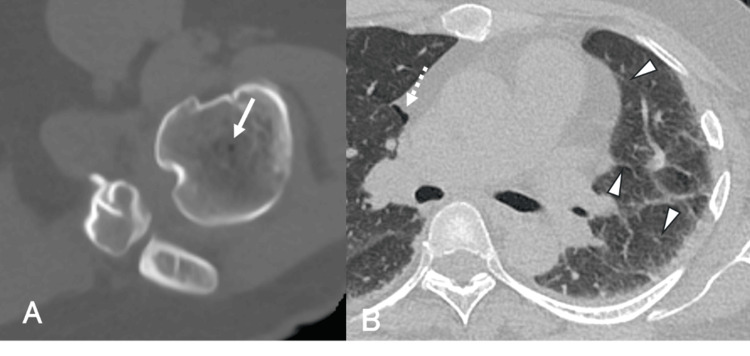
Case 3 Pre-hyperbaric oxygen therapy (HBOT) plain computed tomography (CT) images. A: Bone-window settings (window width of 3000 HU and a window level of 400 HU). B: Lung-window settings. An intramedullary air bubble is observed in the left proximal humerus (solid arrow in A), and a mediastinal bubble is observed on the right side (dashed arrow in B). Thickening of the interlobular septa in the left lung field suggests pulmonary congestion (arrowheads in B).

CT (Figures 3A-3B) demonstrated gas in the mediastinum, intrahepatic portal vein, right femoral vein, bilateral iliopsoas muscles, left sternoclavicular joint, bilateral shoulders, and bilateral hip joints. Intramedullary gas was observed in both humeri. Interlobular septal thickening was suggestive of pulmonary congestion. Post-HBOT CT scans were not performed.

Clinical Course

HBOT was administered according to Treatment Table 6 of the U.S. Navy Diving Manual. The dizziness and nausea resolved by day 2, and HBOT was completed after seven sessions.

## Discussion

The diagnosis of DCS is primarily based on clinical history and symptoms, and treatment focuses on oxygen administration and early HBOT. At our institution, chest CT is routinely performed before HBOT to rule out pneumothorax, pulmonary emphysema, or pulmonary cysts, with head or abdominal CT performed depending on the symptoms. This report summarizes three cases of acute DCS in which intramedullary gas was incidentally identified on pre-HBOT CT. In this series, the severity of DCS was categorized based on the functional involvement of organ systems rather than traditional numerical typing (type Ⅰ/Ⅱ). According to the criteria proposed by Mitchell [1], these cases were classified as "mild" as they presented only with localized musculoskeletal pain or mild vestibular symptoms without evidence of neurological deficits, respiratory distress, or hemodynamic instability.

Imaging findings of acute DCS commonly demonstrate gas in the joints, mediastinum, and mesentery [2]. In severe cases, gas can be observed in the spinal canal, portal vein, and cerebral ventricles [3]. Intramedullary gas associated with acute DCS has been demonstrated in animal models [5,6] and in pathological examinations of fatal DCS cases [7]; however, clinical reports are limited and typically involve severe presentations. Our literature search of the PubMed electronic database using the keywords "intramedullary bubble," "intramedullary gas," and "marrow bubbles" revealed only CT findings reported by Jitsuiki et al. [4] and magnetic resonance imaging (MRI) findings reported by Stéphant et al. [8]. In clinical practice, imaging studies are often not performed in patients with mild DCS. Even when imaging is obtained, intramedullary gas may be regarded as clinically insignificant and therefore not emphasized or reported. Consequently, similar findings may have remained under-recognized.

Despite the relatively mild symptoms, all three patients demonstrated intramedullary gas in addition to articular or venous gas. It is important to note that intramedullary gas on CT is not specific to DCS and can be observed in other conditions. Potential differential diagnoses include emphysematous osteomyelitis, trauma or iatrogenic causes following orthopedic procedures, vacuum phenomenon related to degenerative changes, post-dislocation gas, and gas associated with barotrauma. Based on the diving history, timing of symptom onset, and absence of trauma or infection, alternative diagnoses were deemed unlikely, suggesting that the intramedullary gas observed in these cases may represent a manifestation of DCS. Gas bubble formation in joints is thought to result from supersaturation of inert gases within synovial fluid and periarticular tissues, which are characterized by relatively low perfusion and slow gas washout. Mechanical stress and joint movement may further promote the nucleation and expansion of gas bubbles, leading to the musculoskeletal symptoms characteristic of DCS.

Adult long bones predominantly contain fatty marrow, in which nitrogen is relatively soluble, and marrow perfusion is low. Accordingly, although direct evidence is limited, the mechanism of intramedullary gas bubble formation may share similarities with that of intra-articular gas bubble formation.

Moreover, the clinical significance of intramedullary gas in acute DCS remains unclear. In Case 1, the intramedullary gas resolved after HBOT, and none of the three patients developed complications during short-term follow-up. Conversely, persistent bubbles after HBOT [9] and cases of asymptomatic osteonecrosis [10] have been reported, and data are limited on how intramedullary bubbles influence symptoms or treatment responsiveness. The incidence of decompression-related osteonecrosis among professional divers is high (70.6%) [11], and intramedullary gas has been hypothesized to cause venous stasis, thrombosis, and ischemia, potentially progressing to osteonecrosis with repeated pressure exposures [12]. Although the relationship between intramedullary gas and chronic dysbaric osteonecrosis has been historically discussed, no evidence currently links transient intramedullary gas observed in the acute phase to subsequent osteonecrosis.

Intramedullary gas may be identified on pre-HBOT CT even in mild cases of acute DCS. However, its diagnostic and prognostic relevance remains uncertain, and no conclusions have been drawn regarding its association with chronic osteonecrosis. Long-term imaging follow-up, including MRI to detecct of asymptomatic osteonecrosis, was not performed because all patients returned home after acute treatment, which is an important limitation of this study. Further case and longitudinal studies are needed to elucidate the frequency, mechanisms, and clinical relevance of intramedullary gas in DCS.

## Conclusions

Intramedullary gas observed on CT is not specific and requires consideration of other differential diagnoses, but it can be detected even in patients with mild DCS. Its prognostic significance remains uncertain, and further case reports and longitudinal studies with long-term imaging follow-up are needed to clarify its frequency, underlying mechanisms, and clinical implications in DCS.

## References

[1] Mitchell SJ (2024). Decompression illness: a comprehensive overview. Diving Hyperb Med.

[2] Vann RD, Butler FK, Mitchell SJ, Moon RE (2011). Decompression illness. Lancet.

[3] Wen WC, Tsai MJ, Wu RC (2013). Decompression illness with extensive gas bubble formation. Intern Med.

[4] Jitsuiki K, Kushida Y, Nishio R, Yanagawa Y (2021). Gas in joints after diving: computed tomography may be useful for diagnosing decompression sickness. Wilderness Environ Med.

[5] Smith KH, Stegall P (1974). Experimentally induced osteonecrosis in miniature swine. Proc Symp Dysbaric Osteonecrosis.

[6] Lehner CE, Adams WM, Dubielzig RR, Palta M, Lanphier EH (1997). Dysbaric osteonecrosis in divers and caisson workers. An animal model. Clin Orthop Relat Res.

[7] Kawashima M, Torisu T, Kamo Y, Hayashi K, Kitano M, Tokufuji S (1977). Bone lesions in the femur heads of four autopsy cases of acute decompression sickness. Orthop Traumatol.

[8] Stéphant E, Gempp E, Blatteau JE (2008). Role of MRI in the detection of marrow bubbles after musculoskeletal decompression sickness predictive of subsequent dysbaric osteonecrosis. Clin Radiol.

[9] Dapena JC, Lansdorp CA, Mitchell SJ (2020). Persistent extravascular bubbles on radiologic imaging after recompression treatment for decompression sickness: a case report. Diving Hyperb Med.

[10] Gempp E, Louge P (2005). Early detection of asymptomatic dysbaric osteonecrosis of the shoulder after type 1 decompression sickness: a case report [Article in French]. Rev Med Interne.

[11] Uguen M, Pougnet R, Uguen A, Loddé B, Dewitte JD (2014). Dysbaric osteonecrosis among professional divers: a literature review. Undersea Hyperb Med.

[12] Hutter CD (2000). Dysbaric osteonecrosis: a reassessment and hypothesis. Med Hypotheses.

